# Prospective randomized study comparing outcome of myocardial protection with Del-Nido Cardioplegia versus Saint Thomas Cardioplegia in adult cardiac surgical patients

**DOI:** 10.12669/pjms.38.3.4730

**Published:** 2022

**Authors:** Muhammad Farhan Ali Rizvi, Syed Muhammad Arslan Yousuf, Attaullah Younas, Mirza Ahmad Raza Baig

**Affiliations:** 1 Dr. Muhammad Farhan Ali Rizvi (FCPS), Assistant Professor, Bahawal Victoria Hospital, Bahawalpur, Pakistan; 2 Dr. Syed Muhammad Arslan Yousuf, House Officer, Bahawal Victoria Hospital, Bahawalpur, Pakistan; 3 Mr. Attaullah Younas (B. Sc. Hons CPT), Clinical Perfusionist, Bahawal Victoria Hospital, Bahawalpur, Pakistan; 4 Mr. Mirza Ahmad Raza Baig (B. Sc. Hons CPT), Clinical Perfusionist, Clinical Perfusionist, Hail Cardiac Center, Hail, Saudi Arabia

**Keywords:** Del-Nido Cardioplegia, Myocardial protection, St. Thomas cardioplegia

## Abstract

**Objectives:**

To compare the effectiveness of Del-Nido cardioplegia as myocardial protective agent with Saint Thomas cardioplegia in adult cardiac surgical patients.

**Methods:**

This prospective randomized study was conducted in cardiac surgery department of Bahawal Victoria hospital Bahawalpur, from October 2020 to March 2021. Eighty adult patients who underwent primary Isolated coronary artery bypass grafting (CABG) or isolated Valve surgery requiring cardiopulmonary bypass were randomly divided into Del Nido (DN, n=40) and Saint Thomas (ST, n=40) groups. Data regarding operative and post-operative variables such as cardiopulmonary bypass (CPB) and aortic cross clamp (AXC) times, inotropic requirements, resumption of sinus rhythm, need for electrical defibrillation, post-operative CKMB, blood requirement and ICU stay were noted.

**Results:**

CPB and AXC times were statistically insignificantly different. Resumption of Sinus rhythm was seen significantly in more patients of DN group (95%) than in ST group (72.5%) [p-value 0.05]. Less patients of DN group (5%) were candidates of electrical defibrillation than ST group (17.5%) [p-value <0.001). Post- operative CKMB values were significantly lower in DN group as compared to ST group (30.5±22.6 IU vs 50.5±50.28 IU, p value.008). Blood transfusion was significantly lower in DN group; 50% versus 80% in ST group (p-value 0.005). Ventilation time was significantly less in DN group than ST group (165.95±48.09 minutes versus 165.95±48.09 minutes respectively, p-value 0.03). While ICU stay was also less in DN group; 5.2±0.8 days versus 6.05±1.6 days in ST group (p-value 0.003).

**Conclusion:**

Del-Nido cardioplegia is a reliable and better myocardial protective agent than Saint Thomas cardioplegia in adult cardiac surgical procedures.

## INTRODUCTION

Considering extreme vulnerability of tender myocardium to damage by myocardial reperfusion injury (MRI) during cardiac surgical procedures, multitude of myocardial preservation techniques have been proposed during past few decades with varying degree of success.^1^ The broad categorization of these strategies is based on (i) time of utilization (ii) the manner by which they offer protection to myocardium and (iii) their final destination of action. Ischemic conditioning (pre & post) and hypothermia are examples of first two categories, respectively. Techniques protecting myocytes or non-myocytes are examples of last categorey.^2^

A Cardioplegia solution, representing the preservative technique that protects myocytes, offers many advantages in adult cardiac surgery while it is a mandatory requirement in pediatric cardiac surgery now a days.^3^ Del Nido (DN) cardioplegia solution is a relative recent addition to this legion in adult cardiac surgery. The addition of some novel chemical agents like xylocaine, magnesium sulphate, sodium bicarbonate and mannitol were done on some specific background. The polarizing Xylocaine extremely slows down electrical activity across the cell membrane of myocytes. This not only reduces the intracellular accumulation of calcium, the spearhead of cellular injury, but also reduces energy consumption. Magnesium competes with the calcium entrance in the cell, enhancing its depletion inside the cells. The solution is administered in 1:4 dilution i.e. 20% blood and 80% cardioplegia. Though this dilution effectively reduces hemodilution but low oncotic pressure may result in myocardial edema. Mannitol here rescues myocardium by producing osmotic diuresis along with its role as effective scavenging agent for reactive oxygen species (ROS).^4^

Myocardial preservation is judged by various methods i.e. resumption of normal sinus rhythm after removal of cross clamp, post–operative inotropic requirements, postoperative myocardial enzyme (CKMB or troponin I levels) assay and need to defibrillate the heart after declamping.^5^ Ejection fraction cannot be used a parameter for determining myocardial preservation as it is always overestimated in regurgitant Valvular lesions and unreliable in mixed stenotic and regurgitant Valvular lesions and it is undeniably reduces after surgery in these condition thus jeopardizing the results of research. Comparing DN cardioplegia solution to other solutions in pediatric population has produced mixed results. Some consider DN solution non- inferior to other types of cardioplegia solutions while other have reported lower troponin release, less myofibrillar disarray and less need to defibrillate the heart post-declamping in DN group versus St. Thomas (ST) cardioplegia group.^4^-^7^ Some even have reported more lactate levels and poor anti-inflammatory response in DN group than St. Thomas group.^8^

Having experienced leisure of surgery in single dose cardioplegia and avoidance of harmful effect of repeated dose of cardioplegia, many retrospective and observational studies have evaluated role of DN solution in adult cardiac surgery with mixed results but very few randomized studies have been done. Even worse, locally published studies about DN solution are almost negligible.^9^,^10^ We have done this study to evaluate the safety, efficacy and reliability of DN solution versus ST cardioplegia solution in adult cardiac surgical procedures.

## METHODS

This prospective randomized study was conducted in cardiac surgery department of Bahawal Victoria Hospital Bahawalpur from October 2020 to March 2021. All the patients of age group 20 to 81 years undergoing simple coronary artery bypass grafting (CABG), single vale replacement or double valve replacement (DVR) were included. Patients undergoing simple adult congenital cardiac surgical procedures i.e., Atrial septal defect (ASD) were also incorporated. All patients with re do surgery, emergency surgeries, patients having BMI >30Kg/m^2^, end stage liver or kidney dysfunction, very low EF (<25%), having recent MI or complication of MI were excluded.

Informed consent was taken from patients. Approval letter from Ethical Review Committee, Quaid e Azam Medical Collage was obtained. (Ref No: 1048/DME/QAMC Bahawalpur). Two groups were made by randomly dividing 80 patients in two groups, Del nido (DN) group and St. Thomas (ST) group, 40 patients were placed in each group.

All patients were anesthetized by the same anesthetist following the same technique. Patients were operated through median sternotomy and routine cardiopulmonary bypass (CPB) technique was used. DN cardioplegia was prepared by adding 26 ml Kcl,16 ml H_2_CO_3_ ,16 ml Mannitol, 4-mg MgSO_4_ and 6.4 ml Xylocaine (2%w/v) into 1000ml of cold normal saline. It was delivered in 4:1 (crystalloid: blood) ratio, with induction dose at 20 ml/kg as a single dose or 1000 ml maximum in adults at 4 C. Maintenance dose (10 ml/kg) was not required in a single patient. ST cardioplegia was prepared by adding six cardioplegia ampules (Howard´s solution) in 1000 ml of 0.9% saline. Each ampule (10ml) contains K^+^ 16 mmol/1.19 g, Cl^-^ 49 mmol /3.25 g,Mg^+2^ 16 mmol/1.19g and procaine 1 mmol/27g.The solution was administered in 1:4(crystalloid :blood) ratio, at induction dose of 15-20 ml/kg, at 4C. The maintenance dose at a rate of 10 ml/kg was repeated after every 20-25 minutes.

After removal of AXC, resumption of spontaneous rhythm and need for electrical defibrillation was noted. On completion of procedure patients were weaned off from CPB successfully. Inotropic support initially started was dobutamine, nor-adrenaline/adrenaline was added as double support and Milrinone was added as triple support. Patients were shifted in ICU as intubated, sedated and relaxed and were extubated on fulfilling the requirements of intensivist. Post-operatively creatinine kinase myocardial band (CK-MB) levels were collected 5-6 hours after surgery. Blood transfusions and ICU stay were also recorded. All measurements were recorded on preformed Performa.

By using SPSS version-20, values were displayed in terms of combination of mean and standard deviation for quantitative values and percentages for qualitative values. The continuous variables are analyzed by using Student t test in both the groups and chi-square test for categorical variables for both groups. Fisher exact test and Wilcoxon rank sum test were adopted as appropriate for clinical and abnormal values. Statistically significance is denoted by value of p≤0.05.

## RESULTS

We have studied 40 patients in each of two groups. Del-Nido group [DG] consisted of 32 (80%) patients of CABG, while 8 (20%) patients had Mitral stenosis/regurgitation who underwent Mitral valve replacement. While ST group had 31(78%) patient of CABG and 9(22%) of Mitral valve disease showing same disease pattern in both groups. Almost same demographic characteristics [age, BMI, and hemoglobin] in each of the groups except gender, depicting statistically significant difference (p=05) are shown in Table-I.The risk factors of CAD (hypertension, DM, smoking and COPD) were not deviated and showed insignificant statistics [p>05]. Table-II:

**Table I:** Demographic characteristics.

Variables	Del-Nido	St-Thomas	P-Value
Age [Years]	49±13.79	40.67±16.27	0.09
Gender [Male/Female]	28(70%)/14(30%)	20(50%)/20(50%)	0.05
BMI	22.18±3.98	23.41±3.26	0.13
Hemoglobin[gram/dL]	11.2±1.6	10.5±1.4	0.39

**Table II:** Risk factors of coronary artery disease.

Variables	Del-Nido	St-Thomas	P-Value
Diagnosis [IHD/MS]	32[80%]/8[20%]	31[78%]/9[22%]	0.61
Operation [CABG/MVR]	32[80%]/8[20%]	31[80%]/9[22%]	0.61
HPT [Yes/No]	26[30.2%]/14[16.3%]	29[33.7%]/ 11[12.8%]	0.63
Diabetes [Yes/No]	27/13	34/6	0.06
Smoker [Yes/No]	28/12	34/6	0.18
COPD (Yes/No)	31/9	30/10	0.50
EF%	56.75±11.67454	59.5±10.4	0.97

COPD; chronic obstructive pulmonary disease, EF; ejection fraction, HPT; hyperparathyroidism.

CPB and aortic cross clamp (AXC) times were statistically insignificant [p>0.05] as valued of 88.8±27.1 min and 73.55± 23.2 minutes in DN group as compared to 67.4±24.38 minutes and 64.55±23.6 min in ST group respectively. Statistical similar results [p>0.05] were also observed [Table-III] when we gauze on post CPB values of hemoglobin, urea and serum creatinine. Sinus rhythm was present in more patients (95%) of DN group than in ST group (72.5%) with p value 0.05. Less patients of DN group (5%) were candidates of electrical defibrillation to revert post CPB fibrillation than ST group (17.5%) with statistically significant value of <0.001. Post- operative CKMB values were lower in DN group (30.5±22.6 IU) as compared to ST group (50.5±50.28 IU) leading to the significant p value (0.008) [Table-III]. Blood transfusion was significantly reduced in DN group in comparison to ST group (50% vs 80%, p 0.005) Significant statistical patterns of ventilation time and ICU durations had been recorded among DN group and ST group which were 165.95±48.09minutes and 5.2±.8 days as compared to 203.62±76.87 minutes & 6.05±1.6 days where the p values for former was 0.03 and for later was 0.003. Table-III

**Table III:** Operative and post-operative variables.

Variables	Del-Nido	St-Thomas	P-Value
CPB Time [min]	88.8±27.1	67.4±24.38	0.15
X-clamp Time [min]	73.55±23.2	64.55±23.6	0.84
Hb [Perioperative (gm/dL)]	9.62±1.61	8.96±1.4.1	0.34
Sinus Rhythm [Yes/No]	38(95%)/2(5%)	29(72.5)/11(27.5%)	0.05
Defibrillation [Yes/No]	2(5%)/38(95%)	7(17.5%)/33(82.5%)	<0.001
Inotropes [Single/Double/Triple]	13/22/5	21/14/5	0.12
Blood Transfusion [No/Yes]	20[50%]/20[50%]	8[20%]/32[80%]	0.005
Urea	36.47±12.788	48.9±15.17	0.28
Creatinine	.97±.3	1.04±.28	0.78
CKMB [IU]	30.5±22.6	50.5±50.28	0.008
Ventilation Time [Min]	165.95±48.09	203.62±76.87	0.03
ICU Stay [Days]	5.2±.8	6.05±1.6	0.003

CPB; cardiopulmonary bypass time, CKMB; creatinine kinase myocardial band, ICU; intensive care unit.

## DISCUSSION

We did this study with initial idea that DN cardioplegia solution is at least non inferior to conventional ST blood cardioplegia in terms of myocardial preservation and is more user friendly for both Perfusionist and surgeon in adult cardiac surgery. The results have shown that DN not only is safer and easier to use, it has also proven itself a better myocardial preservative agent than the conventional blood cardioplegia solution. Though many animals, pediatric and adult human studies were present which consolidated our results, scarcity of prospective randomized studies at national level leaded us to perform this study.^10^,^11^

Prolonged AXC (>90 minutes) and CPB times are associated with increased morbidity and mortality and may prolong the ICU and hospital stay.^4^ Both times were usually associated with complex congenital cardiac surgeries or adult surgeries involving re- operations. We did not found statistically significant difference between values of both times among the DN and ST group. Reasons being exclusion of complex congenital and adult re- operations and performance of surgical procedures by surgeon of equal capabilities. Vistarini et al., Guajardo et al., and Kim et al., had also shown statistically insignificant results in adults cardiac procedures when AXC and CPB times were considered.^12^-^14^ Although, Yerebakan et al. and Mick et al. found statistically significant lower values of both times in DN group, nevertheless, former included only the isolated coronary while latter included only isolated valvular patients.^15^,^16^

Recovery of normal sinus rhythm, lack of need to electrically defibrillate the heart after CPB and reduced chances of post -operative dysrhythmias are reflected by maintenance of excellent hemostasis of sodium and calcium by the cardioplegia solution. Xylocaine, a potent sodium (Na^+^) channel blocker served the purpose along with magnesium, a direct calcium (Ca^+2^) antagonist, both being integral components of DN solution.^17^ Inhibition of intracellular accumulation of Na^+^ concomitantly comes with inhibition of Na^+^- Ca^+2^ and Na^+^-H^+^ antiorters, thereby reducing intracellular calcium and intracellular acidosis thus effectively ameliorating chance of myocardial injury and future dysrhythmias. Our study showed that DN solution was clearly ahead of its competitor in these aspects of myocardial preservation as more patients returned in sinus rhythm (p value .05) and less patient required electrical defibrillation (pvalue.00) after removal of cross clamp. Kim et al. and Loberman et al. also showed similar results as ours.^14^,^18^

Post-operative cardiac enzyme assay (CKMB) shows a statistically significant lower values (p value .008) in DN group than ST group. This effect can be explained in many ways. As temperature decreases, viscosity of blood increases and this more viscous blood cardioplegia (administered in 1:4, crystalloid: blood mixture) does not distribute equally in microvasculature of heart specifically in patients of coronary artery disease thus jeopardizing myocardial preservation. The final hematocrit of DN (administered in 4:1, crystalloid: blood mixture) solution is 6-7% as compared to 26-32% of ST, thus former is far less viscous than the latter at same temperature and distributes evenly overcoming above mentioned disadvantage.^19^ Xylocaine, because of its sodium channel paralyzing action, and, magnesium being the direct calcium antagonist, restricts entrance of calcium inside the cells preventing myocardial necrosis. Sodium bicarbonate neutralizes the intracellular acidosis produced by anaerobic glycolysis during periods of arrest. Mannitol reduces the myocardial edema induced by low oncotic pressure of DN solution and it effectively scavenges the reactive oxygen species produced during period of reperfusion. The observation of Voolran et al. and Guajardo et al. are in accordance with our results.^13^,^20^ However, a study at Brigham and Women hospital showed that addition of Xylocaine increased 4-5 times increased CKMB values but their immediate and 1-year mortality was similar between both groups.^21^

The ventilation time was lower in DN group to the extent of statistical significance (p value 0.03) than the ST group. Results of Vistarini et al. and Sorabella et al., were in coherence with our observations.^12^,^22^ Inotropic requirement showed no significant difference between the groups. Results of studies of Yammine and et al., Sorabella et al, and Ota et al., also showed that inotropic requirement was a statistically insignificant variable while comparing DN cardioplegia and other types of cardioplegia solutions.^21^-^23^

Post -operative ICU stay was significantly lower in DN group (p value of .003) but both groups had same mortality. A recently published meta-analysis by Li et al, also demonstrated shorter ICU and shorter hospital stay. They concluded shorter stay to be the direct result of reduce CPB times and less hemodilution offered by DN cardioplegia than others.^24^

One of the most important restraints for adaptation of newer techniques in economically challenged countries like Pakistan is cost effectiveness. DN solution is advantageous is this respect. It’s all ingredients are cheap and easily available and can be made even at time of operation. While other single shot cardioplegia solutions i.e., Custodial are much more expensive and custom made thus reducing its availability.^4^

### Limitations of the study

There were some limitations of this study. The results couldn’t be applied to generally prevalent surgical pattern, as, many studies had compared DN solution to various other solutions, Buckberg, HTK, Custodial and St Thomas and not to a single solution. Similarly, long term effects of increased CKMB levels in control groups could not be ascertained. Effect of DN solution on complex procedures like valve + CABG, emergency surgeries and re do surgeries could not be substantiated. Lack of standardization in usage of inotropic support because of inclination of a surgeon to a specific agent was also a limiting factor. Sample size was small and, finally, failure to incarnate the effects of DN solution over AXC greater than two hours was also an unavoidable confounding factor.

## CONCLUSION

Having observed safer, easier and reliable use of Del-Nido cardioplegia in comparison to St. Thomas cardioplegia solution we recommend more liberal use of the solution in adult cardiac procedures. Nevertheless, a large-scale trial is warranted to further affirm our results in this population.

### Authors’ Contribution:

**MFAR** conceived, designed, writing, editing of manuscript and is responsible for interity of the study.

**SMAY and AY** did data collection and did review.

**MARB** did statistical analysis, did review and final approval of manuscript.
